# Municipal Solid Waste Incineration Fly Ash: From Waste to Cement Manufacturing Resource

**DOI:** 10.3390/ma16062538

**Published:** 2023-03-22

**Authors:** Cristina Marieta, Alexander Martín-Garin, Iñigo Leon, Ana Guerrero

**Affiliations:** 1 Department of Chemical and Environmental Engineering, Faculty of Engineering of Gipuzkoa, University of the Basque Country UPV/EHU, Plaza Europa 1, 20018 Donostia-San Sebastián, Spain; 2 Department of Architecture, University of the Basque Country UPV/EHU, Plaza Oñati 2, 20018 Donostia-San Sebastián, Spain; 3 The Eduardo Torroja Institute for Construction Sciences (IETcc–CSIC), Serrano Galvache s/n, 28033 Madrid, Spain

**Keywords:** clinker reduction, global warming potential, municipal solid waste, incineration technology, fly ash, life cycle assessment, heavy metals

## Abstract

This study investigates the possibility of using municipal solid waste incineration fly ash as a supplementary cementitious material to replace part of the clinker in cement. Life cycle assessment has shown that the partial replacement of clinker with blast furnace slag (CEM III) reduces cement’s global warming potential by ~30%, while replacing clinker with fly ash reduces it by up to 55%. When using CEM III as the control binder in cement in which 55 wt% of the clinker was replaced with hydrothermally treated fly ash, the flexural strength decreased by ~60% and the compressive strength by ~65%. When the fly ash was mixed with calcined and vitrified demolition materials, flexural strength decreased by ~30% and compressive strength by ~50%. The hardening of the hydraulic binders fixed the heavy metals in the municipal solid waste incineration fly ash.

## 1. Introduction

Construction is one of the most highly polluting sectors and is therefore the subject of much research [1,2,3]. Buildings are responsible for one third of all waste generated and constitute one of the heaviest and most voluminous waste streams in the European Union [4]. Furthermore, by volume the building materials industry uses 40% of all material resources and is responsible for 33% of all human-induced emissions [5]. In particular, the most widely used construction material, ordinary Portland cement (OPC), global production of which is estimated at about 0.5 tonnes per person per year [6], is associated with emissions of large quantities of greenhouse gases and environmental pollutants [7]. OPC is essentially a material called clinker, which is obtained from the calcination of limestone with silica, aluminium and iron oxides and blended with small proportions of gypsum or similar sulphates as the setting and hardening time regulator. OPC’s biggest environmental impact derives from the CO_2_ produced during the calcination of limestone [8] and it has been acknowledged that reducing clinker lowers OPC’s global warming potential (GWP). Applicable regulations admit cements in which part of the clinker is replaced with alternative materials, known as supplementary cementitious materials (SCM), such as silica fume and coal fly ashes with pozzolanic activity. Under the EN 197-1:2011 standard, cement types are classified into five groups ranging from CEM I to CEM V. OPC is categorized as CEM I and contains the highest proportion of clinker. As has been demonstrated by life cycle assessment (LCA), cements that contain a large proportion of by-products, such as SCM (e.g., CEM II or CEM III), have the lowest unit emissions of CO_2eq_, these being up to 66% lower per tonne than for CEM I [6,9].

Municipal solid waste (MSW) is likewise a major environmental concern. Incineration is considered to be the most effective treatment technology applied to MSW [10,11], as it can reduce its mass by ~70% while generating a considerable energy output, of which approximately 20–30% can be used for electricity and up to 80% can be used for heating [12]. However, inert residues (25–30% by mass of initial total mass), namely incineration ash (IA), are also generated [13,14]. Based on its collection point and properties, IA is divided into two fractions, known as bottom ash (BA) and incineration fly ash (FA) [15], usually at a mass ratio of 4:1 to 5:1 [16]. The European Waste Catalogue [17] classifies BA as non-hazardous waste since it is not susceptible to leaching beyond the standard regulatory level [18]. BA is typically rich in calcium oxide and silica, so it offers reuse potential as a secondary building material [19]. FA, however, is classified as hazardous waste due to its high heavy metal content; thus, it is not admitted by the applicable regulations [20,21,22,23,24]. As European legislation seeks to promote innovation in recycling [25], considerable efforts are being made to use this residue [26,27,28,29]. This waste also contains Ca, Si and Al, elements that, if appropriately treated in the future, European regulations could admit as SCM [30,31,32,33,34,35]. Moreover, after hardening, the hazardous substances could fix into more stable crystal structures [36,37]. Municipal solid waste incineration fly ash (MSWI FA) also contains high concentrations of chloride. This reduces the capacity of the cement to solidify heavy metals [38] and can lead to critical problems in the cement [39], such as lower compressive strength and durability [40] or aggravation of the corrosion affecting metal bars inserted in concrete [30]. Treatment techniques are employed to perform dichlorination and to eliminate the hazardous elements found in MSWI FA. To achieve dichlorination, simple washing—the most economical option [30,39]—or hydrothermal treatments [39] can be employed. To eliminate the hazardous elements, more complex techniques are used, such as sintering, melting and vitrification [40] or solidification/stabilization [41,42,43]. The effectiveness of hydrothermal treatment has already been analysed, and it was concluded that a hydrothermal treatment (HT-200 °C-1 h) was a useful way to dissolve part of the chloride [44]. In this study, a calcination/vitrification treatment has also been used to research the behaviour of heavy metals in the binders. Thus, the objective in this study was to examine the effectiveness of MSWI FA as a material for use in sustainable cementitious binders (SCBs) intended for the construction sector.

Meanwhile, due to its large specific surface area and high amorphous silica content, silica fume has been used in combination with fly ash from coal combustion as a partial substitute for OPC, showing several advantages in terms of mechanical performance and durability [45]. Heavy metal immobilization also takes place within the hydration phases that occur [46]. Fibreglass (FG) is widely used in the construction sector, so it is increasingly found as another residue in this sector and is practically amorphous silica. Thus, to adjust the Ca/Si ratio in MSWI FA in order to improve mechanical behaviour [47], the latter was mixed with FG residue. In addition, the authors of this study are working on increasing sustainability, specifically via the circular economy, within the university. Thus, measures are being taken to reuse the waste generated during laboratory practicals in the engineering and architecture departments. In this context, mixing cement mortar residue with FG and MSWI FA was also contemplated as another possible alternative to construction demolition waste (CDW). Therefore, hydrothermally treated MSWI FA (referenced as SCM1) and MSWI FA mixed with FG from waste from the manufacture of composites and the residue from mortar specimens (referenced as SCM2) were analysed to study their possible use as SCMs. As CEM III/A is a cement where 36–65% of the clinker has been replaced with blast furnace slag, such as the SCB under study, it was used as control binder. To justify the choice of this cement as reference and the use of MSWI FA as SCM, an LCA focusing on the GWP results was carried out first. Next, physicochemical characterization of the materials and products used in the study was conducted. Finally, the SCBs were prepared by mixing SCM1 and SCM2 with industrial clinker and using gypsum as the setting regulator; the SCB referenced as SCB1 was the binder with SCM1 and that referenced as SCB2 was the binder with SCM2. The main objective of the research was to analyse the feasibility of using an SCM based on MSWI FA as an alternative in the development of an SCB with low environmental impact by reducing clinker content. In this way, the aim is to develop solutions that promote a low-carbon economy, the reuse of materials and the reduction of CDW, thus aligning research with the objectives pursued by the European Union. Furthermore, the behaviour of the heavy metals in the MSWI FA in SCB1 and SCB2 was studied. The results show that the calcination and vitrification treatment is suitable for preparing MSWI FA as a secondary raw material for the production of SCB and that the hardening of hydraulic binders fixes heavy metals.

## 2. Materials and Methods

### 2.1. Materials

The MSWI FA used in this study is produced when waste gases are cleaned and was kindly provided by an MSW incineration plant in Catalonia (Spain). The MSWI FA was hydrothermally treated under the following conditions: (I) 200 mL reactor without stirring; (II) water as a liquid medium, using a water/solid ratio of 10/1; and (III) reactor conditions of 200 °C-1 h (HT-200 °C-1 h) (1.24 MPa pressure). The mixture was then filtered at the end of the HT.

Clinker, gypsum, blast furnace slag (S) and the commercial blast furnace *cement CEM III*/A 42.5N were kindly supplied by a major international cement company; in this cement, between 35% and 65% of the clinker has been replaced with S. Before preparing the binders in the laboratory, both the gypsum and the clinker had to be crushed since they were received in the form of small rocks. For this, firstly a laboratory hammer mill (380 V/50 Hz/three-phase) and then an IKA Werke MF 10 Basic Mill, (IKA, Staufen, Germany) were used. Two experimental SCBs were prepared: SCB1 was prepared by mixing 55 wt% of clinker, 5 wt% of gypsum and 40 wt% of HT MSWI FA (SCM1); while SCB2 was prepared by mixing 55 wt% of clinker, 5 wt% of gypsum and 40 wt% of SCM2, which is obtained by mixing HT MSWI FA (55 wt%) with FG from the composite manufacturing waste (35 wt%) and residue from mortar specimens generated in laboratory practicals (10 wt%). This mixture was calcined at 1000–1050 °C in a muffle furnace to ensure that the SiO_2_ was in the glassy state; the calcined mixture was then cooled with ice. The SCBs prepared as mentioned above underwent grinding in a ball mill to reduce average particle size; obtaining a D_50_ of 12.2 µm for SCB1 and of 9.7 µm for SCB2. A value of 17.8 µm and specific surfaces of 500 m^2^/kg (Mastersizer 3000 particle size analyser, Malvern Panalytical, Malvern, UK) were obtained for CEM III/A. For the study of the mechanical behaviour, mortars were prepared with the binders, standard silica sand and water as per the UNE-EN 196-1 standard in a component ratio by weight of 1:3:0.5. The samples were cured for 1 day, then demoulded and kept in water at 20 °C for 28 days.

### 2.2. Experimental Methods

#### 2.2.1. Environmental Evaluation of the Cements

The environmental evaluation of CEM I was accomplished using a process-based LCA methodology with distinct stages to generate a comprehensive overview of the product’s total environmental effect—goal and scope definition, system boundaries and life Cycle inventory and, finally, data collection and impact assessment method—as defined in ISO 14040, a “cradle-to-gate” LCA. Data collection was carried out at the plant in Añorga, in the Basque Country (Spain), belonging to FYM Heidelberg Cement Group, which kindly provided all the technical data on the process and the product, CEM I 52.5 N [48]. The LCA results were obtained using the Simapro 8.0.1 software and the CML-IA baseline v3.00 method.

##### Goal and Scope

The functional unit was one tonne, since it is recommended in the appropriate Product Category Rules (PCR) and Environmental Product Declaration (EPD), according to ISO 14025.

##### System Boundaries and Life Cycle Inventory

The system used in this study was the production of one tonne of CEM I, which contained 93.5 wt% clinker as the primary reactive compound. The main processes of the system were as follows: (1) the extraction and crushing of raw materials (limestone and calcareous marl), mostly obtained from proprietary quarries; (2) the clinker production, grinding of raw materials and kilning; and (3) the cement production, mixing and grinding with limestone and gypsum. The system extended as far as the factory gate, when the cement was ready for delivery, and only considered storage in the plant’s silos. The limestone quarry was 8 km from the factory and the calcareous marl quarry was adjacent to it. Sand, gypsum and other additions from quarries or providers were a maximum of 200 km from the cement plant; thus, the transport of the raw materials was solely by freight lorry. The primary fuel of the clinker kiln was petroleum coke, although municipal and tyre waste were also burned, which could thus be considered as coke-saving alternatives. Heavy fuel was only used to start the kiln after a technical or scheduled stop. Next, the raw milling, blending and weighing processes were performed. Entry in the clinker kiln was preceded by treatment in a pre-heater cyclone tower, thus helping to save fuel as the raw materials did not enter the kiln cold. The kiln was followed by a grate cooler with bag filters and a heat exchanger. The CEM I cement produced in Añorga (Spain) consisted of 93.5% clinker, 3.5% gypsum and 3% quarry limestone.

##### Data Collection and Impact Assessment Method

According to PCR, data concerning the clinker and cement composition, as well as energy consumption, were also provided by the manufacturer. Likewise, data concerning transport, raw materials, fuel and atmospheric emissions were mostly provided by the producer and normalized for the functional unit. Data concerning infrastructure were taken from the Ecoinvent database. For the electricity used in the cement factory, the national electricity mix was obtained from the evaluation report produced by Red Eléctrica Española (the Spanish electricity grid). The transport of raw materials was appraised in terms of the capacity of the vehicle and the distance travelled. The recycled waste used as alternative fuel (5405 tonnes of municipal and tyre waste) was also considered. According to PCR, the environmental impact categories to consider in LCA are GWP, ozone depletion, acidification of soil and water, eutrophication, photochemical oxidation and abiotic resource depletion (for fossil fuels and for non-fossil resources).

In order to compare the potential environmental impacts of the SCB under study, a simplified LCA model was implemented using the Ecoinvent v3.3 database integrated in the openLCA software 1.10.3.

#### 2.2.2. Physicochemical Characterization

The chemical composition of the raw materials and SCBs used was determined by X-ray fluorescence (XRF). A boron glass bead was prepared for each sample. This was obtained by melting, performed in an induction microwave oven, mixing the Merck Spectromelt A12 flux (ref. 11802, Burlington, MA, USA) and the sample in proportions of approximately 20:1. The chemical analyses of the beads were performed in a vacuum atmosphere using a PANalytical wavelength dispersive X-ray fluorescence (WDXRF) sequential spectrometer (AXIOS model) equipped with an Rh tube and three detectors (gaseous flow, scintillation and Xe sealing). Well-characterized international rock and mineral standards were used to prepare the calibration line. Furthermore, the loss-on-ignition (LOI) of each sample was calculated after subjecting an aliquot part of each one to 1050 °C for one hour in a muffle furnace.

Fourier transform infrared (FTIR) measurements were taken with a Nicolet device (FTIR model), using OMNIC E.S.P. 5.1 software to collect the data. FTIR spectra analysis was performed using the KBr (potassium bromide) disk procedure. Spectra were collected within a scanning range of 450–4000 cm^−1^.

The mineralogical analyses were performed using X-ray diffraction (XRD) with a PANalytical Xpert PRO diffractometer equipped with a copper tube (meanlCuKa = 1.5418 Å, lCuKa1 = 1.54060 Å and lCuKa2 = 1.54439 Å), vertical goniometer (Bragg–Brentano geometry), programmable divergence crack, automatic sample exchanger, secondary graphite monochromator and PixCel detector. The measurement conditions were 40 kV and 40 mA, with a sweep of between 5 and 80 °C. The specific PANalytical Xpert HighScore 1.0 software, combined with the ICDD PDF2 database, was used to process the diffractograms obtained and to identify the existing phases. Analyses were performed on the raw materials used and the anhydrous and 28-day hydrated SCBs.

#### 2.2.3. Study of the Hardened SCBs

The mortars’ mechanical behaviour was analysed via flexural and compressive strength tests performed at 28 days, as per the UNE-EN 196-1 standard, on 40 mm × 40 mm × 160 mm specimens using an Ibertest CIB 200 MDA testing machine (Madrid, Spain). The specimens subjected to the flexural strength test were loaded until failure. The compressive strength test was carried out on the two remaining pieces. Since the porosity of the hardened cement pastes determines their strength and durability, especially in aggressive environments, mercury intrusion porosimetry (MIP) was analysed using a Micromeritics Pore Size 9310 device (Micromeritics, Norcross, GA, United States). Measurements were taken on the hardened SCBs for 28 days and hydration was halted by placing them in ethanol. Thermogravimetric analysis (TG) (TA Instruments Q600 ATD/DSC/TG, New Castle, DE, USA) was also carried out to track the physical and chemical changes with temperature. The instrument was operated from ambient temperature to 1000 °C at a heating rate of 10 °C/min in an N_2_ atmosphere (100 mL/min). Finally, the microstructure was analysed using a Hitachi S-4800 scanning electron microscope (SEM, Tokyo, Japan).

#### 2.2.4. Analysis of Heavy Metals

Quantitative analysis of the heavy metals was carried out using inductively coupled plasma mass spectrometry (ICP-MS). First, analyses of the solid materials—the MSWI FA and SCM2—were performed. Different weights of samples, acids, proportions of acids and microwave cycles were tested until complete digestion was achieved. Digestion was optimized using 50 mg of the sample, 6 mL of 65% nitric acid and 1.5 mL of hydrofluoric acid in a first microwave stage. Subsequently, these same materials were subjected to a second cycle with the addition of 15 mL of saturated boric acid (5%). The Speedwave-four microwave system (Berghof, Eningen, Germany, serial number 5304000) applied a maximum of 120 °C at 80% power. Plasma conditions were as follows: argon gas, PF power 1550 W, RF matching 1.2 V, Smpl Deth 10 mm, nebulizer gas 1 L/min, torch-H 0.2 mm, torch-V 0.7 mm and nebulizer pump 0.1 rps.

A 28-day leaching test was carried out on the hardened binders to study the heavy metals leached out. Adopting the EN 12457-4:2002 standard, 100 g of sample was mixed in 1 L of deionized water and mechanical agitation was maintained for 24 h. Subsequently, the water was analysed by ICP-MS under the conditions described above.

## 3. Results and Discussion

### 3.1. Environmental Evaluation

**Figure 1:**
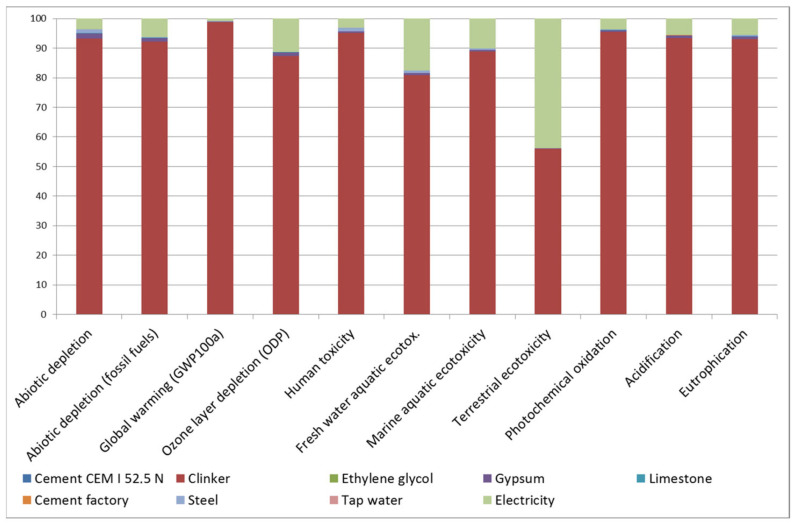
Contribution of each fabrication process in cement manufacture to each impact category.

LCA determined that the CEM I manufactured in the region had a GWP of 748.6 kg CO_2eq_. Analysing the contribution of each fabrication process to each impact category (Figure 1), it is worth noting that clinker is responsible for 80% of the impact in all of them, except for terrestrial ecotoxicity, in which it is responsible for 55%. In that category, high-voltage electricity supply is the second-highest source of impact.

**Figure 2:**
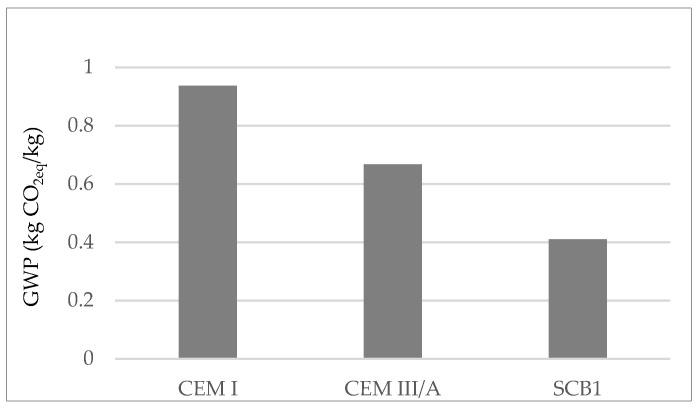
GWP according to LCA of OPC; CEM I, a cement where 35–64% of the clinker is replaced with blast furnace slag; CEM III/A, a simulated cement where 55% of the clinker is replaced with MSWI FA.

It is worth mentioning that the GWP associated with the CEM I assessed is lower than in the literature [49,50]. This is attributed to the fact that the stone extraction quarry is located behind the cement factory, avoiding the significant impact of transport. Therefore, to compare the potential environmental impacts of the binders under study the data from the Ecoinvent v3.3 database for Europe was also used and the compositions of the cements as per EN 197-1 were applied. Figure 2 shows the results in terms of GWP as greatest environmental impact.

The results show that the use of S as SCM reduces the GWP of OPC by approximately 30%, which makes it of interest for use in green concrete structures [51]. With regard to SCB1, it can be seen that the use of MSWI FA as SCM would reduce the GWP of binder manufacture by up to 55%.

### 3.2. Physicochemical Characterization

The chemical compositions of the products (clinker, K), residues (S, GF and OPC mortar), SCMs (SCM1 and SCM2) and anhydrous SCBs were analysed using XRF. Table 1 shows the results for the control cement, CEM III/A 42.5N, of which the SCM is S, SCM1 and SCB1, and SCM2 and SCB2.

Table 1 indicates that CaO and SiO_2_ are the main oxides for clinker. As the lime saturation factor (LSF) is 0.96, in principle it will give rise to an OPC rich in alite. The silica ratio (SR, also known as the silica modulus) is 2.3 and the alumina ratio (AR) is 1.87. These values are in accordance with those for OPC clinker in the literature [52,53].

Concerning the SCMs, it can be seen that SCM1 has an LOI value of 23.96 wt%, a higher value than that found in the literature, which is usually between 10 and 15% [17]. This fact indicates the presence of large amounts of unburnt carbonaceous material. Comparing the XRF results of the MSWI FA without treatment [44], it can be seen that 62% of the chlorines are eliminated after HT, as reported in previous research [54,55]. For SCM2, it should be highlighted that, after calcination, the removal rate of Cl and Na can reach 95% and 94%, respectively, as reported by other authors [56].

The modulus is the characteristic value of each OPC that indicates the relationship between the oxides in percentage terms in order to evaluate the quality of the final cement. The hydraulic modulus (HM = (CaO)/(SiO_2_ + Al_2_O_3_ + Fe_2_O_3_) [57]), the silicate modulus (SM = (SiO_2_)/(Al_2_O_3_ + Fe_2_O_3_) [57]) and the aluminate modulus (AM = (Al_2_O_3_)/(Fe_2_O_3_) [57]) were determined for the SCBs. The results are as follows: HM: 1.8 for CEM III/A, 2.1 for SCB1 and 1.7 for SCB2; SM: 2.5 for CEM III/A, 2.1 for SCB1 and 2.7 for SCB2; AM: 3.1 for CEM III/A, 2.5 for SCB1 and 2.8 for SCB2. The results are within the accepted ranges to obtain cements with optimal properties [58].

**Figure 3:**
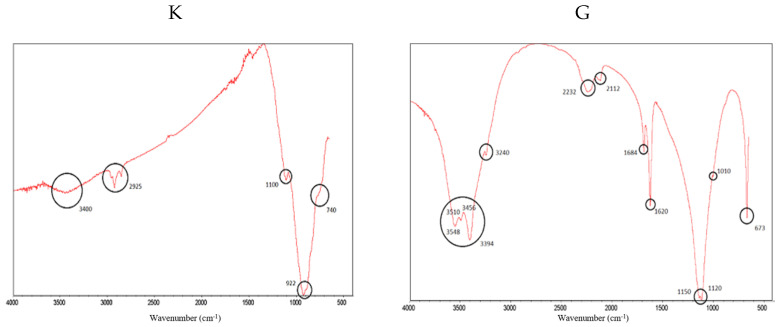
FTIR spectra for clinker (K) (**left**) and gypsum (G) (**right**). The most representative peaks have been highlighted.

Figure 3 shows the FTIR spectra of clinker (K) and gypsum (G), identifying the most important peaks.

In both spectra, vibrations of O–H bonds associated with water are identified between 3548 cm^−1^ and 3240 cm^−1^; for gypsum, this indicates that it is a calcium sulphate dihydrate (CaSO_4_ 2H_2_O) and not anhydrite (CaSO_4_). Thus, ettringite (a hydrous calcium aluminium sulphate mineral) will be formed on SCB hardening [59]. In addition, the sulphate peaks can be seen at 2232 cm^−1^ and 2112 cm^−1^; in the double peak at 1150 cm^−1^ and 1120 cm^−1^, caused by asymmetric “stretching” in the S-O bond; in the shoulder at 1010 cm^−1^; and in the peak at 673 cm^−1^, caused by the “bending” of the S-O bond. The intense peaks at 1684 cm^−1^ and 1620 cm^−1^ are due to the “bending” of the O-H bonds [60,61]. In the clinker spectrum, the peaks at 1100 cm^−1^, 922 cm^−1^ and the shoulder are usually assigned to C2S and C3S in the literature [62,63]. The shoulder at 740 cm^−1^ is associated with C3S [63]. Finally, the peaks around 2925 cm^−1^ are usually attributed to the “stretching” of the aliphatic C–H bonds, which does not make much sense in this sample. However, this fact has already been reported by other authors and has been attributed to a distortion of the technique [64], which we would tend to agree with.

**Figure 4:**
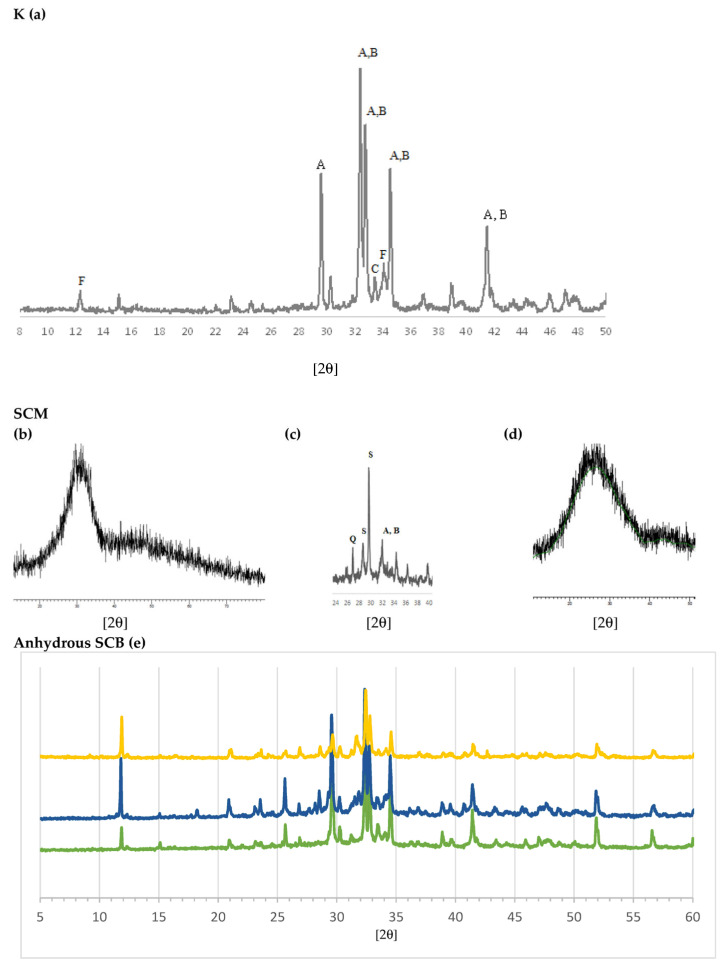
Diffractograms: (**a**) clinker (K), where A is C3S, B is C2S, C is C3A and F is C4AF. SCM: (**b**) blast furnace slag (S). (**c**) SCM2, where A is C3S, B is C2S, Q is quartz and S is silicate. (**d**) Fibreglass (FG). (**e**) Anhydrous SCB: green: CEM III/A; dark blue: SCB1; yellow: SCB2.

The results of mineralogical analysis using XRD are shown in Figure 4. The typical XRD pattern of clinker can be seen in Figure 4a [65]. Match analysis shows that C3S, alite (Ca_3_SiO_5_, hatrurite, A), is the dominant phase. The other major phases—C2S, belite (Ca_2_SiO_4_, larnite, B), C3A, celite (Ca_3_Al_2_O_6_) and C4AF, (Ca_2_(Al,Fe^3+^)_2_O_5_, brownmillerite, F)—overlap each other, especially in the 27–35° 2θ range. The indicated phases explain practically all of the diffraction peaks. The presence of the amorphous phase is also observed as the rise at the bottom of the diagram. Figure 4b–d shows the spectra of the SCMs: S of the CEM III/A and SCM2; the spectrum of the FG also reveals its vitreous character, which makes it of interest for mixing with MSWI FA. As SCM1 was previously analysed by XRD [44], only the S and SCM2 XRD patterns are shown. It can be observed that S is essentially glassy and amorphous, so its diffraction pattern consists of a bottom with a rise or “belly” at 20–35° 2θ. The major crystalline phases identified for SCM1 were the calcite (CaCO_3_), halite (NaCl), sylvite (KCl), anhydrite (CaSO_4_), quartz (SiO_2_), portlandite (Ca(OH)_2_), calcium hydroxychloride (CaClOH), gehlenite (Ca_2_Al_2_SiO_7_) and lime phases (CaO).

The SCM2 was studied before and after calcination. Figure 4c shows the XRD pattern after calcination. In the uncalcined sample, the hydrated cement phase (portlandite and calcite, the so-called “C-S-H gel”) was observed; in the ashes, chlorides, apatite, calcite and quartz were mainly detected; the FG is amorphous, so a rise at the bottom of the diagram was detected. In the calcined sample (SCM2, Figure 4c), the presence of calcium silicates C3S (Ca_3_SiO_5_, hatrurite, A) and C2S, belite, (Ca_2_SiO_4_, larnite, B), silicates such as gehlenite (Ca_2_Al(AlSi)O_7_) and wollastonite (CaSiO_3_) (S), and quartz (SiO_2_, Q) is observed. This suggests that, during calcination at 1050 °C, the CaO of the calcite and portlandite reacted with the silica of the FG and the amorphous part of the hydrated cement (C-S-H gel). During calcination, the chlorides KCl and NaCl volatilize, as also detected by XRF (Table 1).

The diffractograms of the SCBs are shown together for better comparison (Figure 4e). For the CEM III/A (green), silicate phases similar to clinker (a) are identified: C3S (A), C2S (B) and C4AF (F). However, differences are also seen; thus, CEM III/A has more C3S than C2S and more calcite; and traces of quartz (Q) can also be seen. The difference with clinker observed in the 33–34° 2θ range is attributed to differences in the C3A, aluminate, C4AF and ferrite phases, which play a role in the setting and early hardening properties but make a low contribution to the final strength. It can also be seen that the size of the glassy phase is greater than in clinker. Regarding the binders with the MSWI FA-based SCM, it can be seen that both present a complex set of phases. The main differences between them are that, in SCB1, KCl (sylvite) and NaCl (halite) are identified, as shown in the chemical analysis in Table 1. These phases are not observed in the SCB2, which means that they were eliminated after calcination. With respect to SiO_2_, in both SCBs the presence of quartz can be detected; however, in the case of SCB2 a greater presence of glassy material is observed around 25–40° 2θ.

### 3.3. Hardened SCBs

The hardened mass formed by the hydration of common cement pastes is composed mainly of hydrated calcium silicates—denoted by the symbol C–S–H and often called the tobermorite phase because their chemical composition is similar to natural tobermorite (5CaO·6SiO_2_·5H_2_O)—combined with the crystallization of ettringite (Ca_6_Al_2_(SO_4_)_3_(OH)_12_·26H_2_O), calcium hydroxide, Ca(OH)_2_, portlandite (filling pores [66]) and hydroxy aluminate phases, denoted by AF_m_ [67]. At the early ages, a limited amount of C-S-H gel is generated. With ongoing curing, more portlandite is generated, filling the pore structure and improving the mechanical properties. The porosity strongly depends on the w/c ratio, but also on the type of binder [68]. Thus, in this study, to better interpret the mechanical behaviour the porosity of the mortars was determined by MIP. For CEM III/A, the p_MIP_ was 26.4 %vol; for SCB1, it was 18.0 %vol; and for SCB2, it was 24.2 %vol. The results are similar to those found in the literature for common cement mortars [69] and binders containing FA-based SCM [70,71]. The mechanical behaviour was investigated through flexural and compressive strength tests. The flexural strength of the control binder, CEM III/A, was 8.41 ± 0.56 MPa; for SCB1 it was 3.60 ± 0.52 MPa; and for SCB2 it was 5.85 ± 0.24 MPa. For compressive strength, the values obtained were 53.30 ± 1.14 MPa for CEM III/A, 17.98 ± 0.25 MPa for SCB1, and 25.32 ± 0.52 MPa for SCB2. It is worth mentioning that the most porous mortar was the one with the highest mechanical resistance and the least porous was the one with the lowest value. This means that, regardless of the porosity, the microstructure generated in the SCBs does not attain the strength of CEM III/A. Although it is clear that the MSWI FA-based binders cannot be used for structural concretes, the results for SCB2 are within a suitable range of values for use in other types of application, such as the construction of ditches, pavements and similar works, with the important environmental benefits indicated above [72]. Likewise, the presence of vitreous silica improves the strength of MSWI FA-based SCB. It is clear that CEM III/A is not only more environmentally friendly, but it can also be considered a high-strength cement.

**Figure 5:**
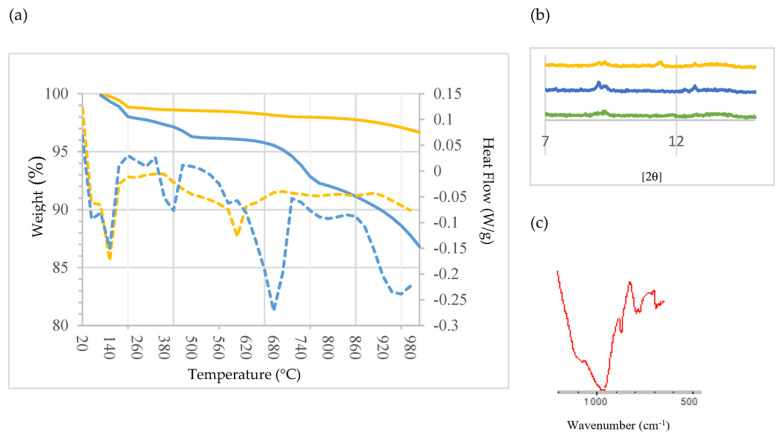
(**a**) TG/DTG analysis of the SCBs: blue: SCB1; yellow: SCB2. Solid line: Weight (%), dotted line: Heat Flow (W/g). (**b**) XRD pattern of CEM III/A (green), SCB1 (blue) and SCB2 (yellow). (**c**) FTIR spectrum of SCB1.

Figure 5a shows the TG curves and their corresponding derivative function (DTG) curves for SCB1 (blue) and SCB2 (yellow). As can be seen, the thermal process can be divided into four stages: from ambient temperature to ~220 °C (stage 1), from ~220 °C to ~500 °C (stage 2), from ~500 °C to ~820 °C (stage 3) and from ~820 °C to 1000 °C (stage 4). The first stage is similar in both binders, featuring two appreciable mass losses: at ~60 °C and ~140 °C. This behaviour has already been observed by other authors [73,74]. The first mass loss is attributed to the dehydration of the ettringite; it can be observed that SCB1 loses more mass than SCB2. The second endothermic peak is attributed to the dehydration of the C-S-H [75]. In the second stage, it can be seen that the SCBs behave differently; thus, the mass loss at ~420 °C, attributed to the decomposition of Ca(OH)_2_ [73], is much more abrupt in the case of SCB1; while for SCB2, the decomposition is much more progressive, so it can be posited that the portlandite in SCB2 is more stable than that in SCB1. It is worth mentioning the small peak observed at 300 °C. Bearing in mind that these SCBs contain some chlorine—1.98 wt% for SCB1 and 2.66 wt% for SCB2 (Table 1)—the presence of Friedel’s salt (Ca_2_Al(OH)_6_Cl_2_ 2H_2_O) could be considered. It was confirmed by analysing the diffractogram of the binders cured for 28 h. As can be seen in Figure 5b, a peak is detected for SCB1 and SCB2 between 11 °C and 13 °C, which is attributed to Friedel’s salt in the literature [74]. Likewise, when performing FTIR, a peak at ~786 cm^−1^ due to an Al-OH bending mode was also observed (Figure 5c). In the temperature range of stage 3, decarbonization of calcium carbonate (CaCO_3_), calcite and carbonated C-S-H gel takes place. The carbonization of C-S-H leads to the formation of an unstable type of calcium carbonate which decomposes between 500 °C and 700 °C [75]. In this case, this is much more pronounced for SCB1 than for SCB2. Finally, in the last stage, above 800 °C, there is a significant loss of mass for SCB1, which is attributed to vaporization of the chlorine compounds present in SCM1 but not in SCM2, as was identified by XRD (Figure 3).

The microstructure of hardened cement is formed in three stages. In the first stage, the resulting Ca(OH)_2_ is present primarily as portlandite, forming hexagonal crystals about 40 microns in size, while the aggregates take the form of columns, being ~25% by volume of the solid phase. The morphology of the portlandite crystals is dependent on the w/c ratio, the type of admixtures and the additives. In the second stage, the first forms of C–S–H are created, these being between 50 and 60% of the volume of all the solid phases. According to the Diamond model [76], at the early stages of hydration fibres from two microns in size are formed, taking the shape of a mesh (the so-called “honeycomb”) and becoming increasingly massive until reaching the characteristic formless massive gel of old pastes. Finally, pore filling of the hardening cement paste by short fibres of ettringite [75], typically formed as elongated crystals similar to needles, takes place in the third stage. These stages range in duration from several days to several months [77].

**Figure 6:**
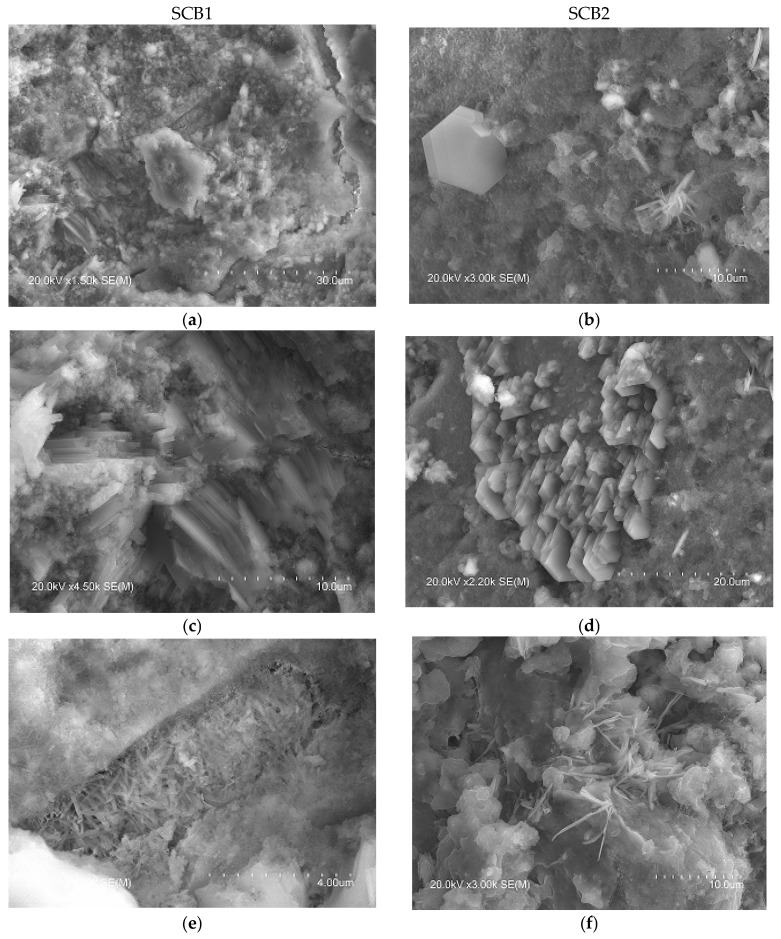

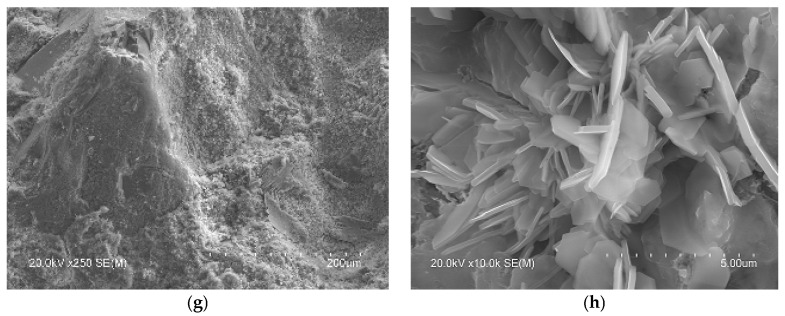
SEM images of the microstructure of the hydrated SCBs at 28 days. Main phases: Left: SCB1: (**a**) portlandite and ettringite, (**c**) portlandite, (**e**) ettringite, (**g**) unhydrated MSWI FA grains; right: SCB2: (**b**) portlandite and C-S-H, (**d**) portlandite, (**f**) ettringite and Friedel’s salt, (**h**) ettringite and Friedel’s salt.

In the microstructure of the SCB2, the typical phases of common cements mentioned above were observed. Thus, Figure 6 shows images with hexagonal plates of portlandite (b), where the C-S-H “honeycomb” structure can also be seen, along with column aggregates of portlandite (d) and short fibres of ettringite (f). Examination of this bundle of fibres, shown in Figure 6h, at a higher magnification reveals crystals with a similar shape to those observed by other authors, attributed to Friedel’s salt [74,78,79]. In the microstructure of SCB1, although no hexagonal plates of portlandite were observed, some microstructures, which could be aggregates of portlandite (lower left corner of images (Figure 6a,c)), are visible. In this SCB1 matrix, microcracks were observed with elongated crystals similar to needles inside (the right-hand side of images (Figure 6a,e)). These crystals are attributed to ettringite [77]. The presence of these microcracks may explain the low mechanical strength values obtained in mortars based on this SCB. In the literature, these cracks observed in binders based on waste incineration ash are attributed to the metallic Al components that evolve into hydrogen gas during curing [17]. Likewise, it could be thought of as an excess of sulphoaluminate, as seen in TG/DTG. Furthermore, round particles that are attributed to unhydrated MSWI FA grains (Figure 6g) were observed. This microstructure can explain the low strength values of this binder. Moreover, as can be seen in images Figure 6a,b, the microstructure of hardened SCB2 is denser than that of SCB1 which, keeping in mind that the w/c ratio and the 28 days of curing are the same for both systems, indicates a higher amount of C-S-H in SCB2.

### 3.4. Behaviour of Heavy Metals in MSWI FA

The major heavy metals in MSWI FA were Zn and Pb, which had a mass concentration of 5099.1 mg/kg^−1^ and 1153.5 mg/kg^−1^, respectively. The content of other heavy metals was relatively low: Cu (679.4 mg/kg^−1^), Cr (165.6 mg/kg^−1^), Cd (44.2 mg/kg^−1^) and Ni (92.5 mg/kg^−1^) [47]. After calcination and vitrification (SCM2) there was a significant decrease, the new values being Zn (2515.8 mg/kg^−1^), Pb (48.2 mg/kg^−1^), Cu (112.3 mg/kg^−1^), Cr (193.1 mg/kg^−1^), Cd (2.6 mg/kg^−1^) and Ni (51.6 mg/kg^−1^). This was especially noteworthy in the case of Cd and Pb, since 95% of these metals were eliminated; the 85% reduction in Cu was also significant. This behaviour was attributed to the high volatilization of Cd and Pb. With regard to zinc, it has been found in the literature that, with a thermal treatment, up to 82% can be eliminated [56], although in this case only 50% was removed. The reason for this could be attributed to the fact that the volatilization of heavy metals depends on many factors, the chlorine content being one of them, and, although a large part of the chlorine was eliminated, the trace that remained could have an impact on the volatilization of this metal.

A 28-day leaching test was carried out on the hardened binders to study the heavy metals leached out. Heavy metals of the filtrate were determined by ICP-MS and the results are shown in Table 2.

Although immobilization in the hardened binders (SCB1 and SCB2) is not total for some metals (e.g., Cr), the concentrations are below the highest permissible values according to the Urban Waste Water Treatment Directive [80,81]. Due to the differences observed between SCM2 and the binders, it can be concluded that the heavy metals were fixed during hardening. Therefore, these results not only prove that the hardening of hydraulic binders fixes heavy metals, but also that the calcination and vitrification treatment is suitable for preparing MSWI FA as a resource for the production of construction binders.

## 4. Conclusions

The aim of this paper has been to investigate MSWI FA-based SCMs for use as a secondary raw material in the production of SCBs. The behaviour of the heavy metals in the MSWI FA has also been studied. Based on the results obtained, the following conclusions can be drawn:

- The use of MSWI FA-based SCMs could reduce the cement’s GWP by up to 55%.
- The main elements in MSWI FA are calcium, silica, alumina and iron, a composition similar to that of the mineral admixture used in cement-based materials. However, large amounts of chloride and traces of heavy metals are also detected, which means the ashes must be pre-treated before use as SCMs. HT is a useful way to dissolve part of the chloride, while the heavy metals can be eliminated by calcination/vitrification.
- The main minerals present in the SCMs are quartz, gehlenite, portlandite and calcite, the major minerals of raw materials for binders used in construction.
- The presence of vitreous silica improves the mechanical behaviour of the hardened HT MSWI FA-based SCBs, increasing compressive strength by ~30% and flexural strength by ~40%.
- Mortars of HT MSWI FA-based SCBs present a similar microstructure to those of OPC. Thus, hexagonal plates of portlandite, a C-S-H “honeycomb” structure and short fibres of ettringite are generated during the hydration process. When the only SCM in the SCB is HT MSWI FA microcracks are formed that explain the low mechanical strength values of the hardened SCB. These cracks are attributed to the metallic Al components that evolve into hydrogen gas during the curing process. When HT MSWI FA is mixed with calcined and vitrified demolition materials a densification of the mortar takes place and the mechanical behaviour improves.
- TG/DTG, DRX, FTIR and SEM confirmed the presence of Friedel’s salt, a stable aluminate phase whose composition is sensitive to the local chemical environment and with which, in the presence of chloride, an ion exchange takes place. Thus, Friedel’s salt acts as a “sink” for chloride ions and thereby retards diffusion of it.
- The heavy metals in MSWI FA are fixed during the hardening of the SCB.

### Recommendations for Further Research

Future work includes research of mixtures of MSWI FA and kaolinitic clay, which, after a suitable calcination heat treatment, turns into metakaolin with high pozzolanic activity. On the other hand, once the possible treatments applied to MSWI FA for its use as an SCM have been investigated, the objective of the research will be to develop geopolymer or alkali-activated materials using this residue. In particular, utilization of these developments in 3D printing is considered due to the ability of this technology to produce concrete elements with complex shapes and significant environmental benefits.

## Figures and Tables

**Table 1:** Chemical compositions of SCMs and SCBs (wt%), as determined by XRF.

Sample	SiO_2_	Al_2_O_3_	Fe_2_O_3_ *	MnO	MgO	CaO	Na_2_O	K_2_O	TiO_2_	P_2_O_5_	SO_3_	Cl	LOI **
K	19.70	5.37	2.86	0.04	1.41	61.23	0.10	0.89	0.22	0.33	2.01	0.14	1.55
S	35.47	9.42	0.28	0.11	7.56	41.82	0.01	0.45	0.69	0.00	1.75	0.10	1.11
GF	50.22	12.72	0.23	DL ^1^	0.41	20.92	0.55	0.19	0.08	0.02	0.06	0.18	5.44
PC	8.02	2.01	1.11	0.02	0.73	46.48	DL	0.37	0.10	0.02	0.85	DL	37.60
CEM III	21.13	6.26	2.04	0.06	2.74	53.53	0.10	0.73	0.33	0.12	3.66	0.25	5.80
SCM1	17.22	5.34	1.10	0.04	2.00	30.95	9.84	1.77	1.10	1.22	1.11	3.32	23.96
SCB1	15.74	5.40	2.15	0.05	1.84	48.47	0.93	1.26	0.62	0.78	5.07	1.98	13.94
SCM2	33.69	9.27	1.33	0.03	1.66	40.45	0.57	0.26	0.93	0.95	3.26	0.35	3.72
SCB2	21.73	6.03	2.12	1.48	1.48	50.76	1.07	1.07	0.48	0.57	4.51	2.66	7.72

* The iron content has been expressed as total Fe_2_O_3_; ** LOI: loss-on-ignition, after subjecting an aliquot part of each one to 1050 °C in a muffle furnace for one hour; ^1^ DL: detection limit.

**Table 2:** Leaching concentration of heavy metals.

Sample	Cr (μg/mL)	Ni (ng/mL)	Cu (ng/mL)	Zn (ng/mL)	Cd (ng/mL)	Pb (ng/mL)
SCM2	2.9	70.9	232.9	84.1	1.7	ELQ
SCB1	0.3	ELQ	15.5	40.6	ELQ	ELQ
SCB2	0.1	ELQ	ELQ	ELQ	ELQ	ELQ

For the quantification of the samples, a calibration has been used in concentrations of 1–500 ng/mL. The estimated limits of quantification (ELQ) for Ni, Cu, Zn, Cd and Pb were 1, 2, 10, 1n and 1 ng/mL, respectively.

## Data Availability

Not applicable.

## Abbreviations

AM	aluminate modulus
AR	alumina ratio
BA	bottom ash
CDW	construction and demolition waste
CEM I	cement classification of Portland cement without any main addition
CEM III/A	commercial blast furnace cement
DL	detection limit
DTG	derivative function TG curves
ELQ	estimated limit of quantification
EPD	environmental product declaration
FA	fly ash
FG	fibreglass
FTIR	Fourier transform infrared
GWP	global warming potential
HM	hydraulic modulus
HT	hydrothermal treatment
IA	incineration ash
ICP-MS	inductively coupled plasma mass spectrometry
LCA	life cycle assessment
LOI	loss-on-ignition
LSF	lime saturation factor
MIP	mercury intrusion porosimetry
MSW	municipal solid waste
MSWI FA	municipal solid waste incineration fly ash
MIP	porosity by MIP
OPC	Portland cement
PCR	product category rules
S	blast furnace slag
SCB	sustainable cementitious binder
SCB1	the binder with SCM1
SCB2	the binder with SCM2
SCM	supplementary cementitious material
SCM1	hydrothermally treated MSWI FA
SCM2	MSWI FA with FG from the manufacture of composites and CDW
SEM	scanning electron microscope
SM	silicate modulus
SR	silica modulus
TG	thermogravimetric analysis
WDXRF	wavelength dispersive X-ray fluorescence
XRD	X-ray diffraction
XRF	X-ray fluorescence
